# Clinical Values of Long Non-coding RNAs in Bladder Cancer: A Systematic Review

**DOI:** 10.3389/fphys.2018.00652

**Published:** 2018-05-30

**Authors:** Guoming Su, Qili He, June Wang

**Affiliations:** ^1^Department of Pharmacy and Laboratory, Sichuan Nursing Vocational College, Chengdu, China; ^2^Institute of Toxicological Detection, Sichuan Center for Disease Control and Prevention, Chengdu, China; ^3^Guangdong Provincial Key Laboratory of Medical Molecular Diagnostics, Guangdong Medical University, Dongguan, China

**Keywords:** bladder cancer, long non-coding RNAs, clinicopathological parameters, prognosis, diagnosis, systematic review

## Abstract

**Background:** Increasing evidence shows that dysregulated expression of long non-coding RNAs (lncRNAs) can serve as diagnostic or prognostic markers in bladder cancer. The aim of this study was to evaluate the clinical values of dysregulated lncRNAs in bladder cancer.

**Methods:** Eligible studies were systematically searched in PubMed, Embase, and Web of Science databases from inception to December 2017. Odds ratios (OR) were calculated to investigate the correlation between lncRNAs and clinicopathological parameters. Pooled hazard ratios (HR) and 95% confidence interval (CI) were calculated to explore the prognostic value of lncRNAs in bladder cancer. Pooled diagnostic parameters were also calculated to estimate the performance of lncRNAs in diagnosing bladder cancer. All statistical analyses were performed by using STATA 13.1 program.

**Results:** A total of 37 relevant studies were included to the present systematic review according to the inclusion and exclusion criteria, including 26 on clinicopathological parameters, 19 on prognosis, and 7 on diagnosis. For clinicopathological parameters, MALAT1 expression was significantly associated with lymph node metastasis (OR = 2.731; 95% CI: 1.409–5.292; *p* = 0.003), and high-level expression of XIST was related to larger tumor size (OR = 2.473; 95% CI: 1.159–5.276; *p* = 0.019) and higher TNM stage (OR = 0.400; 95% CI, 0.184–0.868; *p* = 0.020). For the prognostic values, the most significant association was observed between increased expressions of SPRY4-IT1 and poor overall survival (OS) (HR = 3.716; 95% CI: 2.084–6.719; *p* < 0.001); high MALAT1 expression was significantly associated with poor OS (HR = 1.611; 95% CI: 1.076–2.412; *p* = 0.020). For the diagnostic values, UCA1 expression profile achieved a combined AUC of 0.92, with sensitivity of 0.84 and specificity of 0.89 in distinguishing patients with bladder cancer from non-cancerous controls.

**Conclusions:** In summary, systematic review elaborated that abnormal lncRNAs expression can serve as potential markers for prognostic evaluation in bladder cancer patients. In addition, the diagnostic meta-analysis concluded that abnormally expressed UCA1 can function as potential diagnostic markers for bladder cancer.

## Introduction

Bladder cancer ranks as the ninth most frequently-diagnosed cancer worldwide and it is estimated that nearly 500,000 cases are diagnosed annually worldwide (Antoni et al., 2017). Despite improvements in current clinical treatment such as surgery, radiation therapy, and chemotherapy, 50–70% of patients are relapsed within the next 5 years (Terracciano et al., 2017). Therefore, it is urgent to find novel markers for diagnosis at early stage and identify effective therapeutic targets for improving the survival rate of patients with bladder cancer.

Long non-coding RNAs (lncRNAs) are generally defined as RNA transcripts longer than 200 nucleotides that lack an open reading frame. Recently, increasing evidences show that lncRNAs play important roles in various cancers, which influence all the “hallmarks of cancer” (Gutschner and Diederichs, 2012). It is reported that lncRNAs are involved in various cell biological processes, such as tumor cell proliferation, apoptosis, invasion, and metastasis (Hansji et al., 2014; Terracciano et al., 2017). So the aberrant expression patterns of lncRNAs are correlated with cancer diagnosis and prognosis and serve as predictors of patient outcomes. For example, LncRNA H19 expression was up-regulated and closely related to TNM cancer stages in patients with gastric cancer, which can serve as a potential non-invasive diagnostic biomarker in gastric cancer (Hashad et al., 2016). Sun et al. (2016) indicated that the lncRNA antisense non-coding RNA in the INK4 locus (ANRIL) was up-regulated in colorectal cancer tissues, which was associated with the survival rate of patients with colorectal cancer. LncRNA-activated by TGF-β (lncRNA-ATB) was significantly up-regulated in hepatocellular carcinoma metastases and associated with poor prognosis (Yuan et al., 2014).

Up to now, it was reported that some lncRNAs was aberrantly expressed in bladder cancer, such as HULC (Wang J. et al., 2017), MALAT1 (Li et al., 2017), and SNHG16 (Cao et al., 2017). Hu R. G. et al. (2017) found that the lncRNA cancer susceptibility candidate 8 (CASC8) was significantly down-regulated in bladder cancers and associated with the advanced stage of bladder cancer patients, overexpression of which remarkably suppressed the bladder cancer cell proliferation. Hepatocellular carcinoma up-regulated long non-coding RNA (HULC) promoted bladder cancer cells proliferation and inhibited apoptosis via regulation of ZIC2 and PI3K/AKT signaling pathway (Wang J. et al., 2017). LncRNA urothelial cancer-associated 1 (UCA1) promoted bladder cancer cell migration and invasion via hsa-miR-145/ZEB1/2/FSCN1 pathway (Xue et al., 2016). Recently several studies have investigated the prognostic and diagnostic value of lncRNAs in bladder cancer. However, most studies examining the clinical values of aberrantly expressed lncRNAs was limited by the small sample size or a single lncRNA. Therefore, we conducted a systematic review and meta-analysis to evaluate the clinicopathological, prognostic, and diagnostic roles of multiple lncRNAs expression in patients with bladder cancer.

## Methods

### Publication search

The present systematic review was performed according to the PRISMA Statement (Moher et al., 2010) (see Table S1 PRISMA Checklist) and Cochrane Collaboration guidelines (http://handbook.cochrane.org/). We searched the Pubmed, Embase and Web of Science to identify relevant studies until December 21, 2017. The search strategies were based on combinations of the following key words: (“long non-coding RNA,” “lncRNA,” “lincRNA,” “long ncRNA,” “long intergenic non-coding RNA”) AND (“bladder”) AND (“cancer,” “carcinoma,” “neoplasm,” “tumor,” “tumors,” “tumor,” “tumors,” “malignancy,” “metastasis”). In addition, the references of eligible studies and relevant systematic reviews were checked for other eligible studies. We provided the detailed search strategies and results in the Table S2.

### Selection criteria

The included studies met the following criteria: (1) patients in the study were diagnosed with bladder cancer; (2) studies investigated the association between lncRNAs and bladder cancer; (3) sample size was no less than 40 cases; (4) for clinicopathological studies, the correlation between lncRNAs and clinicopathological parameters of patients with bladder cancer was performed, and the expression level of lncRNAs was divided into high or low groups; (5) for prognostic studies, the correlation between lncRNAs and survival was performed and the primary endpoints as overall survival (OS), disease free survival (DFS), cancer-specific survival (CSS) or recurrence-free survival (RFS) were clearly defined, then Kaplan–Meier survival curves or sufficient original data was provided to extract hazard ratio (HR) with 95% confidence interval (CI); (6) for diagnostic studies, diagnostic accuracy of lncRNAs for bladder cancer was performed, and sufficient data was provided for constructing the diagnostic two-by-two tables.

The exclusion criteria were: (1) overlapping or duplicate data; (2) lack of essential information; (3) letter, review article, case report, and conference abstract; and (4) non-English papers and non-human studies.

### Data quality assessment and extraction

Data were extracted by two authors independently from included studies using a predefined data extraction form. Then another author verified them and any discrepancies were resolved by consensus. The following information were collected: (1) basic information: first author's name, publication year, country, study design, patient population, lncRNAs, expression, sample size, tumor type, detected sample, detection method, and cutoff value; (2) clinicopathological parameters: gender, age, tumor size, tumor number, histological grade, TNM stage, tumor stage T, and lymph node metastasis; (3) prognostic information: follow-up months, outcome of survival analysis, and HR with corresponding 95% CI; (4) diagnostic information: sensitivity, specificity, area under the curve (AUC), sample sizes for diagnostic analysis, and data for two-by-two tables [true positive (TP), false positive (FP), true negative (TN), and false negative (FN)]. For studies that showed only Kaplan-Meier survival curve, HR with their 95% CI was calculated by using Engauge Digitizer version 4.1 and Tierney's method (Tierney et al., 2007).

We assessed the methodological quality of prognostic studies using the Newcastle-Ottawa Scale (NOS) tool that was extracted and modified from previous studies (Gao et al., 2017). The NOS scores ranged from 0 to 8, and a study with the higher scores indicated better methodological quality. Moreover, the Quality Assessment of Diagnostic Accuracy Studies (QUADAS) list was used to systematically assess the quality of all the included diagnostic studies (Whiting et al., 2003). Fourteen items from the QUADAS list were applied to each article, with an answer of “yes,” “no,” or “unclear.” The answer “yes” obtained a score of 1, whereas “no” or “unclear” gained a score of 0, and the full score was 14. If a cumulative score is higher than 8, the study will be considered as low risk of bias.

### Statistical analysis

Heterogeneity among the included studies was assessed by using the Cochrane's *Q*-test and *I*_2_ statistics. If heterogeneity (*p* < 0.05 or *I*_2_ > 50%) was statistically significant among studies, the random-effect model was chosen for the meta-analysis; otherwise, the fixed-effect model was used. Odds ratio (OR) with 95% CI was used to evaluate association between lncRNAs expression and clinicopathological parameters. Pooled HR with 95% CI was calculated to summarize the effect between lncRNAs and survival in patients with bladder cancer. For the diagnostic meta-analysis, correlated diagnostic accuracy indexes were computed as follows: sensitivity, specificity, positive likelihood ratio (PLR), negative likelihood ratio (NLR), diagnostic odds ratio (DOR), summary receiver operating characteristic (SROC) curve, and AUC. Publication bias was detected using Deeks' regression test of asymmetry (Deeks et al., 2005). All statistical analyses were performed by using STATA 13.1 program (Stata Corpotion, College Station, Texas, USA).

## Results

### Study selection

**Figure 1:**
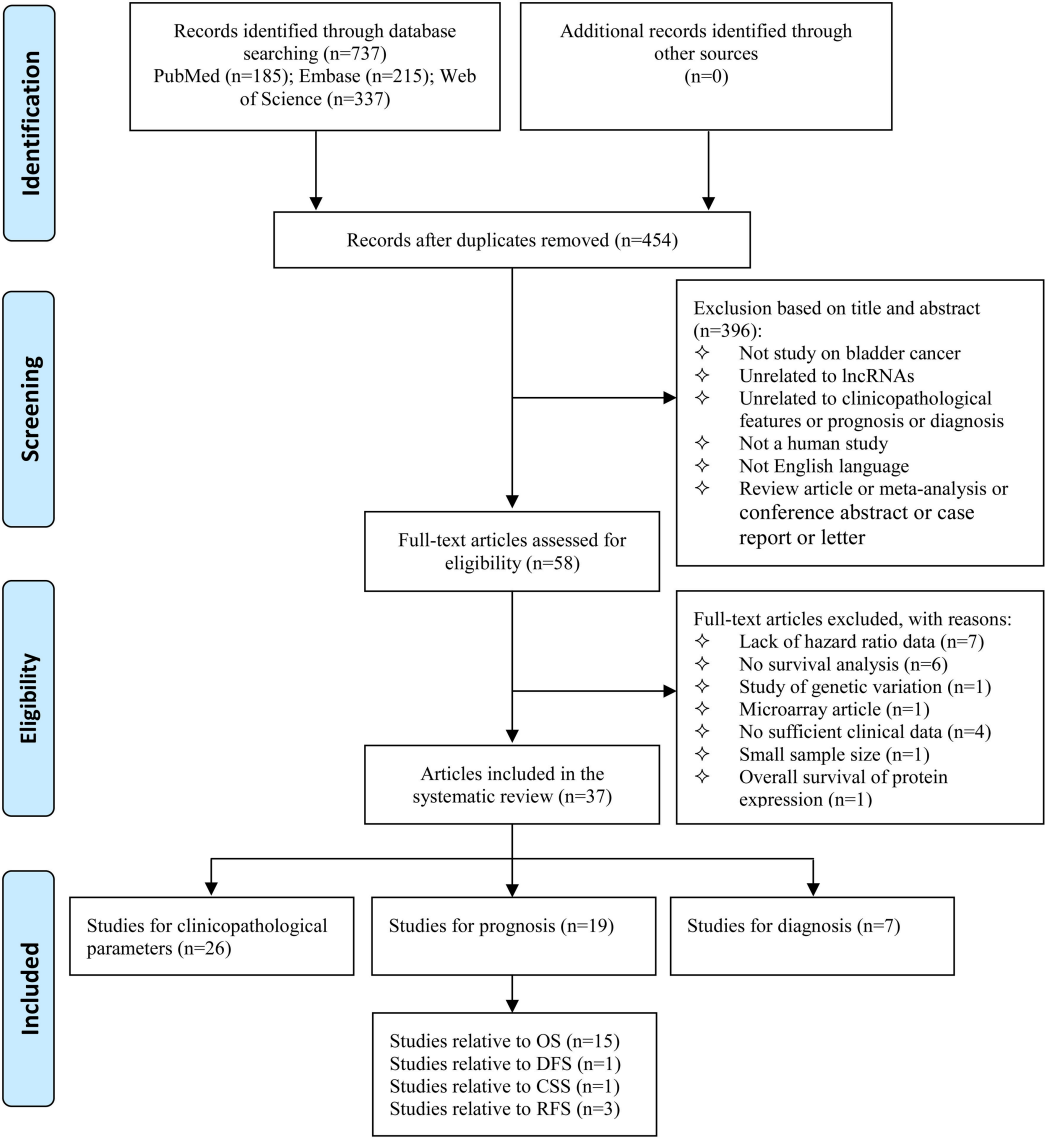
Flow diagram of study selection process. lncRNAs, long non-coding RNAs; OS, overall survival; DFS, disease free survival; CSS, cancer-specific survival; RFS, recurrence-free survival.

As shown in the flow diagram (Figure 1), we identified 737 records in the electronic databases, including Pubmed, Embase, and Web of Science. Firstly, 283 duplicate records were excluded using EndNote X8. With the inclusion and exclusion criteria, 396 records were excluded by reviewing titles and abstracts. Subsequently, the 58 remaining full-text articles were assessed. Among 58 articles, 21 were excluded from the quantitative synthesis for the reasons depicted in Figure 1. No additional studies were identified by a manual search of the references of the original studies. Finally, the remaining 37 articles were eligible for the systematic review (Wang et al., 2006; He et al., 2013, 2016a,b; Fan et al., 2014; Li et al., 2014, 2017; Srivastava et al., 2014; Yan et al., 2014; Chen et al., 2015, 2017; Eissa et al., 2015a,b; Milowich et al., 2015; Zhao F. J. et al., 2015; Zhao X. L. et al., 2015; Duan et al., 2016; Iliev et al., 2016; Zhan et al., 2016a,b, 2017a,b; Zhang et al., 2016, 2017; Cao et al., 2017; Cui et al., 2017; Dudek et al., 2017; Du et al., 2017; Hu Y. Y. et al., 2017; Liao et al., 2017; Lin et al., 2017; Tolkach et al., 2017; Wu et al., 2017; Xiong et al., 2017; Yang et al., 2017; Ye et al., 2017; Zhuang et al., 2017), including 26 studies for clinicopathological parameters, 19 studies for prognosis, and 7 studies for diagnosis.

### Clinicopathological parameters

Table 1 summarized the main characteristics of studies on the association between lncRNAs and clinicopathological parameters. All the selected studies on clinicopathological parameters were from China, with 20/26 (76.9%) being published between 2016 and 2017. The systematic review of clinicopathological parameters was performed in 1,896 patients with bladder cancer, including urinary bladder cancer, urothelial carcinoma of the bladder, muscle-invasive bladder cancer, bladder transitional cell carcinomas, and non-muscle invasive bladder cancer. Twenty-four lncRNAs were described in the 26 studies involved in clinicopathological parameters. The expression of MALAT1 (Fan et al., 2014; Li et al., 2017), ASAP1-IT1 (Yang et al., 2017), SPRY4-IT1 (Zhao X. L. et al., 2015), lncRNA-n336928 (Chen et al., 2015), linc-UBC1 (He et al., 2013), SUMO1P3 (Zhan et al., 2016b), HNF1A-AS1 (Zhan et al., 2017b), CCEPR (Zhan et al., 2017a), linc00346 (Ye et al., 2017), XIST (Hu Y. Y. et al., 2017; Xiong et al., 2017), ZEB2-AS1 (Wu et al., 2017), ZEB1-AS1 (Lin et al., 2017), PVT1 (Cui et al., 2017), ABHD11-AS1 (Chen et al., 2017), SNHG16 (Cao et al., 2017), lncRNA-UNMIBC (Zhang et al., 2016), PANDAR (Zhan et al., 2016a), AATBC (Zhao F. J. et al., 2015), and HOTAIR (Yan et al., 2014) were up-regulated in bladder cancer patients, while the expression of lncRNA-LET (Zhuang et al., 2017), lncRNA-LOWEG (Liao et al., 2017), NBAT1 (Du et al., 2017), BANCR (He et al., 2016b), and MIR31HG (He et al., 2016a) were down-regulated. Only one study reported that down-regulated lncRNA-LOWEG were significantly associated with gender of patients (Liao et al., 2017). The results of these studies indicated that 24 lncRNAs were not significantly correlated with age of patients and tumor number. Two studies claimed that up-regulated ZEB2-AS1 (Wu et al., 2017) and XIST (Hu Y. Y. et al., 2017) were significantly related to tumor size. Dysregulated SPRY4-IT1 (Zhao X. L. et al., 2015), lncRNA-n336928 (Chen et al., 2015), SUMO1P3 (Zhan et al., 2016b), HNF1A-AS1 (Zhan et al., 2017b), CCEPR (Zhan et al., 2017a), ZEB1-AS1 (Lin et al., 2017), NBAT1 (Du et al., 2017), PVT1 (Cui et al., 2017), ABHD11-AS1 (Chen et al., 2017), PANDAR (Zhan et al., 2016a), AATBC (Zhao F. J. et al., 2015), and HOTAIR (Yan et al., 2014) were significantly associated with histological grade. Dysregulated ASAP1-IT1 (Yang et al., 2017), lncRNA-LET (Zhuang et al., 2017), XIST (Hu Y. Y. et al., 2017; Xiong et al., 2017), ABHD11-AS1 (Chen et al., 2017), SNHG16 (Cao et al., 2017), BANCR (He et al., 2016b), and MIR31HG (He et al., 2016a) were significantly associated with TNM stage. Furthermore, SPRY4-IT1 (Zhao X. L. et al., 2015), MALAT1 (Li et al., 2017), linc-UBC1 (He et al., 2013), lncRNA-LET (Zhuang et al., 2017), ZEB2-AS1 (Wu et al., 2017), XIST (Hu Y. Y. et al., 2017), PVT1 (Cui et al., 2017), and SNHG16 (Cao et al., 2017) were significantly associated with lymph node metastasis status in patients with bladder cancer.

**Table 1:** Summary of the comparison for the *p*-values of the association between lncRNAs and clinicopathological parameters.

**Study**	**Year**	**Country**	**Study design**	**Patient population**	**TNM stage**	**Treatment of patient**	**LncRNAs**	**Expression**	**Case number**	**Cutoff**	***p*****-values**							
											**Gender**	**Age**	**Tumor size**	**Tumor number**	**Histological grade**	**TNM stage**	**Tumor stage T**	**Lymph node metastasis**
Yang L	2017	China	RCS	UBC	I-IV	Radical cystectomy	ASAP1-IT1	Up	58	Median	1.000	0.790	NA	0.150	NA	0.017	0.014	0.160
Li C	2017	China	PCS	BC	NA	Transurethral resection of bladder tumor and radical resection of the bladder	MALAT1	Up	120	Mean	0.769	0.841	0.607	0.755	0.023	NA	0.004	0.009
Zhuang J	2017	China	PCS	UBC	0-IV	Surgery	lncRNA-LET	Down	60	Median	0.146	0.267	NA	0.399	0.424	0.039	NA	0.012
Zhan YH	2017	China	CCS	UCB	NA	Radical or partial cystectomy	HNF1A-AS1	Up	79	Fold-change	0.915	0.597	0.081	0.076	0.008	NA	0.034	0.651
Zhan YH	2017	China	CCS	UCB	NA	Radical or partial cystectomy	CCEPR	Up	55	Fold-change	0.677	0.473	0.768	0.087	0.021	NA	0.014	NA
Ye T	2017	China	CCS	BC	NA	Surgical resection	linc00346	Up	52	NA	0.075	0.634	NA	NA	0.910	NA	0.894	NA
Xiong YY	2017	China	CCS	BC	I-IV	Surgical resection	XIST	Up	67	NA	NA	0.273	0.067	0.901	0.784	0.036	NA	NA
Wu X	2017	China	CCS	BC	NA	Curative resection	ZEB2-AS1	Up	52	NA	0.931	0.337	0.023	NA	NA	NA	0.026	0.048
Lin JH	2017	China	CCS	BC	NA	NA	ZEB1-AS1	Up	55	Fold-change	0.792	0.745	0.940	0.197	0.001	NA	0.027	1.000
Liao XH	2017	China	CCS	UNB	0-IV	Radical cystectomy	lncRNA-LOWEG	Down	55	Relative	0.04	0.58	0.97	NA	0.60	0.18	NA	0.63
Hu YY	2017	China	PCS	BC	I-IV	Resection surgery	XIST	Up	52	Fold-change	0.658	0.540	0.028	0.425	0.036	0.012	NA	0.042
Du D	2017	China	RCS	BC	NA	Surgical resection	NBAT1	Down	79	Fold-change	0.580	0.807	0.179	NA	0.043	NA	0.027	0.073
Cui Y	2017	China	PCS	BC	NA	Surgery	PVT1	Up	146	Mean	0.86	0.31	0.19	0.72	0.02	NA	0.03	<0.01
Chen MW	2017	China	CCS	UC	0-IV	NA	ABHD11-AS1	Up	66	Fold-change	0.575	0.587	NA	NA	<0.001	0.001	0.001	0.663
Cao X	2017	China	PCS	BC	0-IV	Total or partial removal of bladder	SNHG16	Up	46	Mean	1.000	0.568	NA	NA	NA	0.003	1.000	0.000
He AB	2016	China	CCS	BC	0-IV	Surgical resection	MIR31HG	Down	55	NA	0.415	0.607	0.109	NA	0.415	0.010	NA	0.114
Zhan YH	2016	China	CCS	UBC	NA	Radical resections	SUMO1P3	Up	55	Fold-change	0.528	0.580	0.114	0.054	0.005	NA	0.037	0.618
Zhang SM	2016	China	PCS	NMIBC	NA	NA	lncRNA-UNMIBC	Up	75	Fold-change	0.4456	0.9851	0.0712	0.0931	0.0725	NA	0.9652	NA
Zhan YH	2016	China	CCS	UCB	NA	Partial or radical cystectomy	PANDAR	Up	55	Fold-change	0.953	0.745	0.061	0.759	0.010	NA	0.027	0.607
He AB	2016	China	CCS	UNB	0-IV	Radical cystectomy	BANCR	Down	54	Relative	0.09	0.230	0.065	NA	0.194	0.004	NA	0.559
Zhao XL	2015	China	PCS	UCB	NA	Transurethral resection, partial cystectomy and radical cystectomy	SPRY4-IT1	Up	68	Mean	0.707	0.790	0.874	0.813	<0.001	NA	0.017	<0.001
Chen T	2015	China	PCS	BC	NA	Radical cystectomy	lncRNA-n336928	Up	95	Fold-change	0.479	0.656	0.825	0.307	0.002	NA	<0.001	NA
Zhao FJ	2015	China	CCS	BC	NA	Cystectomy	AATBC	Up	90	Fold-change	0.899	0.863	NA	NA	0.010	NA	0.015	0.315
Fan Y	2014	China	RCS	BT	NA	NA	MALAT1	Up	95	Median	0.315	0.237	0.213	NA	0.097	NA	0.104	0.063
Yan TH	2014	China	PCS	BC	NA	Transurethral resection of the bladder	HOTAIR	Up	110	Mean	>0.05	>0.05	>0.05	NA	0.031	NA	>0.05	NA
He W	2013	China	RCS	BC	NA	Surgical resection	linc-UBC1	Up	102	Fold-change	0.655	0.961	NA	NA	0.828	NA	0.422	0.021

*RCS, retrospective cohort study; PCS, prospective cohort study; CCS, case-control study; UBC, urinary bladder cancer; UCB, urothelial carcinoma of the bladder; MIBC, muscle-invasive bladder cancer; BTCC, bladder transitional cell carcinomas; BC, bladder cancer; UNB, urothelial neoplasms of the bladder; UC, urothelial cancer; NMIBC, non-muscle invasive bladder cancer; BT, bladder tumors; LncRNAs, long non-coding RNAs; Up, up-regulation; Down, down-regulation; NA, not available*.

**Figure 2:**
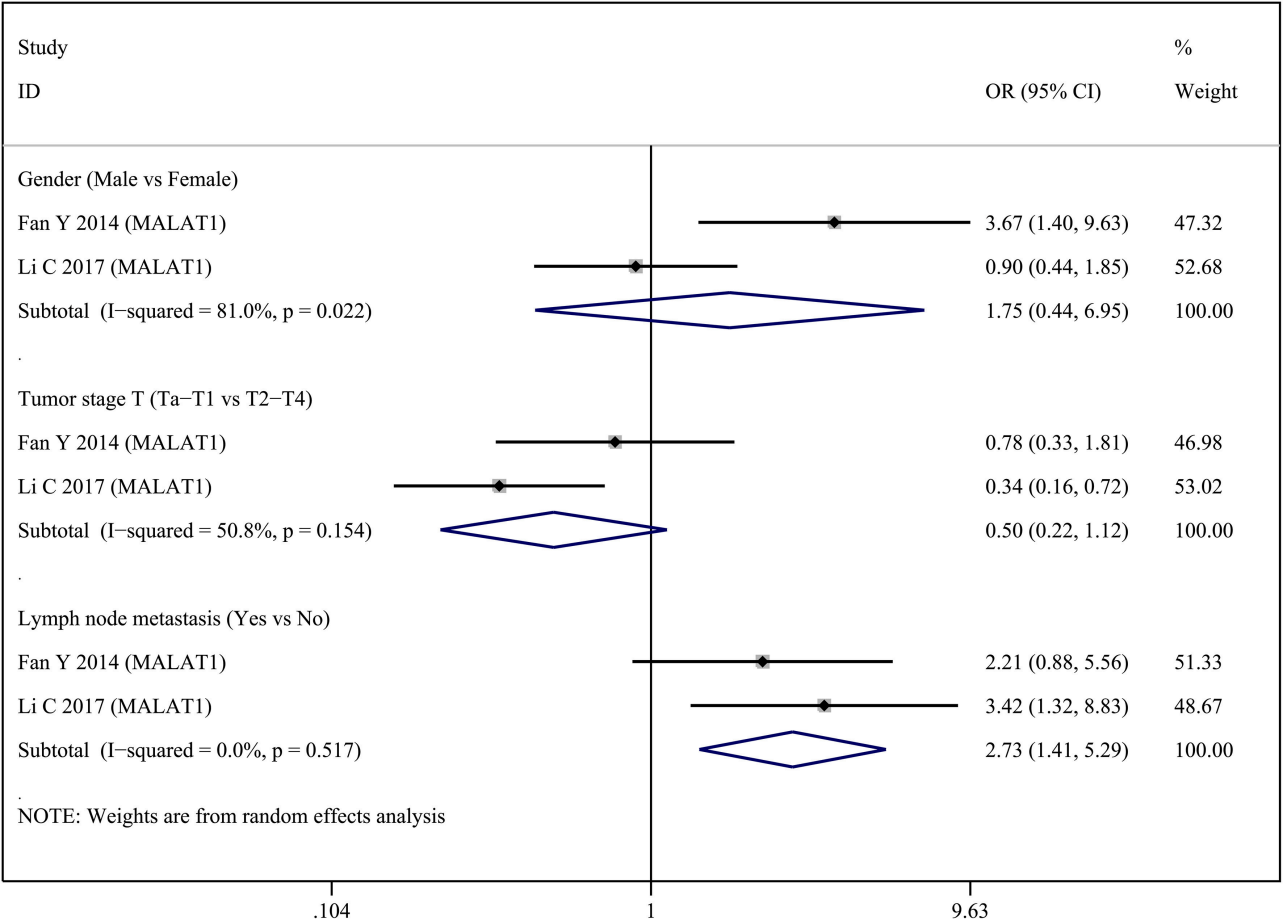
Forest plots of odds ratios (OR) for the association between MALAT1 expression and clinicopathological features in bladder cancer patients.

**Figure 3:**
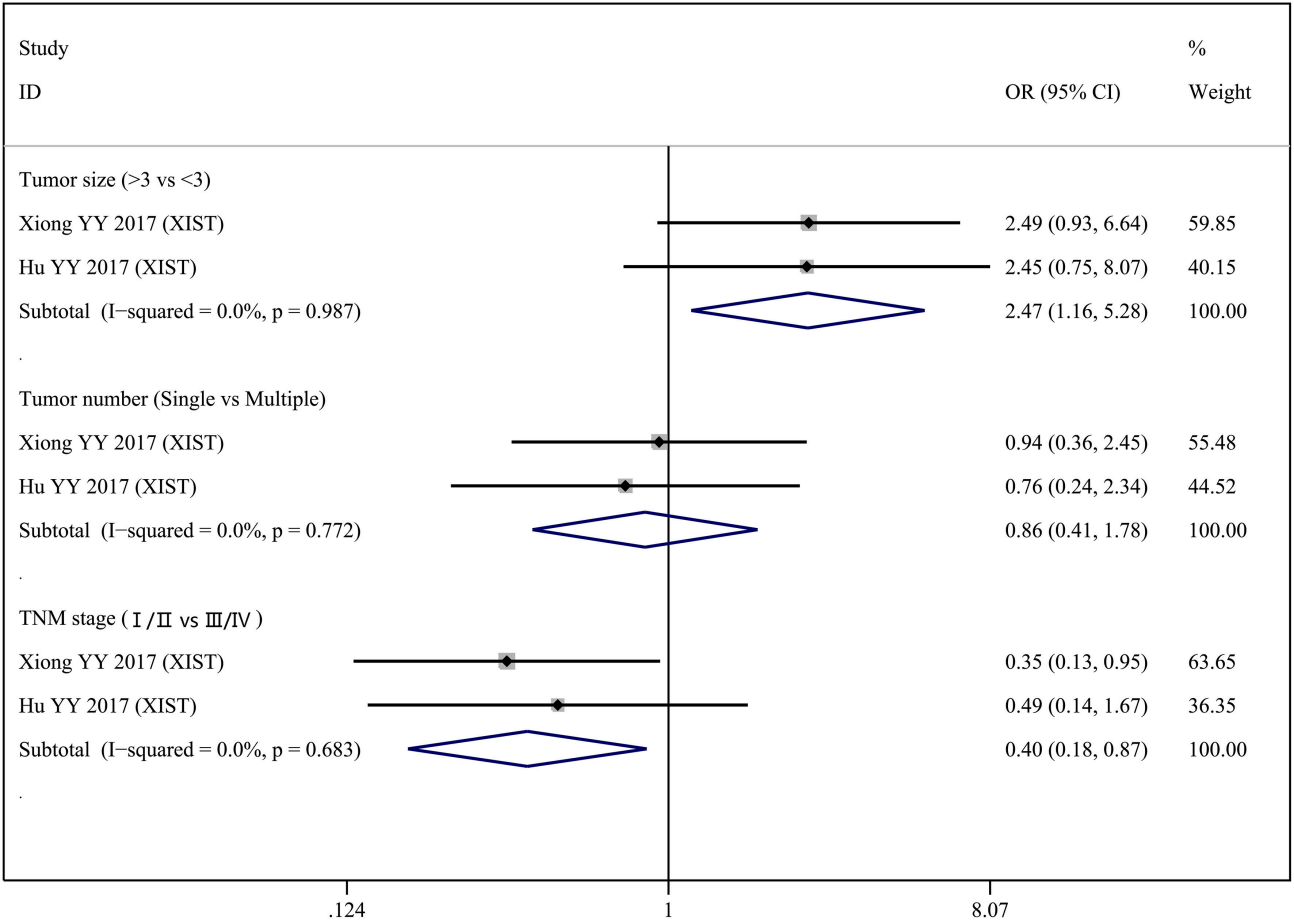
Forest plots of odds ratios (OR) for the association between XIST expression and clinicopathological features in bladder cancer patients.

Two lncRNAs (MALAT1 and XIST) were investigated in two studies, respectively. Therefore, we performed a meta-analysis to evaluate the association between MALAT1 and XIST and clinicopathological parameters. For the MALAT1, we combined two studies with a total of three groups according to different clinicopathological parameters (Figure 2). Heterogeneity was observed in two groups (Gender, *I*_2_ = 81.0%, *p* = 0.022; Tumor stage T, *I*_2_ = 50.8%, *p* = 0.154); therefore, a random effect model was used for the quantitative pooling. The results revealed that high MALAT1 expression was significantly associated with lymph node metastasis (OR = 2.731; 95% CI: 1.409–5.292; *p* = 0.003). However, expression of MALAT1 was not significantly associated with gender of patients (OR = 1.748; 95% CI: 0.440–6.951; *p* = 0.428) and tumor stage T (OR = 0.501; 95% CI: 0.225–1.120; *p* = 0.092). For the XIST, we combined two studies with a total of three groups according to different clinicopathological parameters (Figure 3). After analysis using fixed effect model, our results revealed that high expression of XIST was significantly associated with larger tumor size (OR = 2.473; 95% CI: 1.159–5.276; *p* = 0.019). In addition, high expression of XIST was related to higher TNM stage (OR = 0.400; 95% CI: 0.184–0.868; *p* = 0.020). However, expression of XIST was not significantly associated with tumor number (OR = 0.859; 95% CI: 0.413–1.783; *p* = 0.682). Publication bias was not assessed because only two studies investigated the same lncRNA MALAT1 or XIST that were pooled into the meta-analysis.

### Prognosis

Nineteen studies on prognosis were eligible for the final analysis, with 13/19 (68.4%) being published between 2016 and 2017. Most of the eligible studies were conducted in Chinese populations (84.2%), followed by German (5.3%), Czech (5.3%), and Netherlander (5.3%). Additionally, NOS scores indicated that 17 (89.5%) of the 19 eligible studies were not <7 (Table S3). Summary of lncRNAs used as prognostic biomarkers of bladder cancer was presented in Table 2. The systematic review of prognosis was performed in 1,604 patients with bladder cancer, including urinary bladder cancer, urothelial carcinoma of the bladder, muscle-invasive bladder cancer, bladder transitional cell carcinomas, and non-muscle invasive bladder cancer. Fifteen studies containing 17 lncRNAs were available to evaluate the relationship between abnormally expressed lncRNAs and OS of bladder cancer patients. HRs and their corresponding 95% CI were produced from the eligible studies. An observed HR <1 implied that the patients with increased lncRNAs expression had a better survival. Conversely, an observed HR > 1 implied that the patients with increased lncRNAs expression had a worse survival. Increased expressions of MALAT1 (Fan et al., 2014; Li et al., 2017), ASAP1-IT1 (Yang et al., 2017), SPRY4-IT1 (Zhao X. L. et al., 2015), TUG1 (Iliev et al., 2016), lncRNA-n336928 (Chen et al., 2015), GHET1 (Li et al., 2014), linc-UBC1 (He et al., 2013), YRNA5 (Tolkach et al., 2017), XIST (Hu Y. Y. et al., 2017), PVT1 (Cui et al., 2017), and SNHG16 (Cao et al., 2017) were significantly correlated with poor prognosis in OS, along with decreased expressions of CAT1297 (Dudek et al., 2017), lncRNA-LET, YRNA1 (Tolkach et al., 2017), YRNA3 (Tolkach et al., 2017), YRNA4 (Tolkach et al., 2017), and NBAT1 (Du et al., 2017). It was worth noting that the most significant association was observed between increased expressions of SPRY4-IT1 and poor OS (HR = 3.716; 95% CI: 2.084–6.719; *p* < 0.001). One study containing 4 lncRNAs were available to evaluate the relationship between abnormally expressed lncRNAs and CSS of bladder cancer patients. Increased expressions of YRNA4 (Tolkach et al., 2017) and YRNA5 (Tolkach et al., 2017) were significantly correlated with poor prognosis in CSS, along with decreased expressions of YRNA1 (Tolkach et al., 2017) and YRNA3 (Tolkach et al., 2017). Three studies containing 3 lncRNAs were available to evaluate the relationship between abnormally expressed lncRNAs and RFS of bladder cancer patients. Increased expressions of lncRNA-UNMIBC (Zhang et al., 2016) and HOTAIR (Yan et al., 2014) were significantly correlated with poor prognosis in RFS, along with decreased expression of MEG3 (Duan et al., 2016). It was also worth noting that the most significant association was observed between increased expressions of HOTAIR and poor RFS (HR = 4.712; 95% CI: 2.894–8.714; *p* < 0.001). Only one study was available to evaluate the relationship between abnormally expressed lncRNAs and DFS of bladder cancer patients. Decreased expression of GAS5 (Zhang et al., 2017) was significantly correlated with poor prognosis in DFS.

**Table 2:** Summary of lncRNAs used as prognostic biomarkers of bladder cancer.

**Study**	**Year**	**Country**	**Study design**	**Patient population**	**TNM stage**	**Treatment of patient**	**LncRNAs**	**Expression**	**Number of patients**	**Number of patients**		**Detected sample**	**Detection method**	**Cutoff**	**Follow-up months**	**Survival analysis**	**HR (High vs. Low) (95% CI)**
										**High**	**Low**						
Yang L	2017	China	RCS	UBC	I-IV	Radical cystectomy	ASAP1-IT1	Up	58	29	29	Tissue	qRT-PCR	Medium (1.42)	50 (Total)	OS	Multivariate: 2.639 (1.056–6.579)
Zhang H	2017	China	PCS	BTCC	I-III	Transurethral resection and radical cystectomy	GAS5	Down	82	41	41	Tissue	qRT-PCR	NA	60 (Total)	DFS	Univariate: 0.4824 (0.2865–0.8122)
Li C	2017	China	PCS	BC	NA	Transurethral resection of bladder tumor and radical resection of the bladder	MALAT1	Up	120	64	56	Tissue	ISH	Mean (2.65)	60 (Total)	OS	Multivariate: 2.056 (1.236–3.879)
Dudek AM	2017	Netherlands	RCS	MIBC	NA	NA	CAT1297	Up	121	60	61	Tissue	TCGA	NA	168 (Total)	OS	Multivariate: 0.508 (0.284–0.909)
Zhuang J	2017	China	PCS	UBC	0-IV	Surgery	lncRNA-LET	Down	60	30	30	Tissue	qRT-PCR	Median	60 (Total)	OS	Kaplan-Meier: 0.70 (0.19– 2.57)
Tolkach Y	2017	Germany	PCS	UBC	NA	Transurethral resection or radical cystectomy	YRNA1 YRNA3 YRNA4 YRNA5	Down	88	NA	NA	Tissue	qRT-PCR	NA	Median (range): 51 (1–210)	OS CSS	Multivariate: OS/CSS YRNA1 0.806 (0.357–1.818)/ 0.820 (0.315–2.128) YRNA3 0.699 (0.307–1.613)/ 0.833 (0.308–2.222) YRNA4 0.926 (0.415–2.041)/ 1.111 (0.441–2.857) YRNA5 1.250 (0.617–2.564)/ 1.370 (0.556–3.333)
Hu YY	2017	China	PCS	BC	I-IV	Resection surgery	XIST	Up	52	32	20	Tissue	qRT-PCR	NA	50 (Total)	OS	Kaplan-Meier: 1.51 (0.43–5.24)
Du D	2017	China	RCS	BC	NA	Surgical resection	NBAT1	Down	79	45	34	Tissue	qRT-PCR	NA	60 (Total)	OS	Kaplan-Meier: 0.41 (0.14–1.17)
Cui Y	2017	China	PCS	BC	NA	Surgery	PVT1	Up	146	73	73	Tissue	qRT-PCR	Mean (3.64)	60 (Total)	OS	Multivariate: 2.00 (1.06–3.79)
Cao X	2017	China	PCS	BC	0-IV	Total or partial removal of bladder	SNHG16	Up	46	25	21	Tissue	qRT-PCR	Mean	60 (Total)	OS	Kaplan-Meier: 2.27 (0.43–11.91)
Iliev R	2016	Czech Republic	PCS	MIBC	NA	Partial or radical cystectomy	TUG1	Up	47	26	21	Tissue	qRT-PCR	Mean (0.1232)	Median (range): 30 (12–104)	OS	Multivariate: 2.54 (1.13–5.74)
Zhang SM	2016	China	PCS	NMIBC	NA	NA	lncRNA-UNMIBC	Up	75	45	30	Tissue	qRT-PCR	NA	42 (Total)	RFS	Multivariate: 2.362 (1.504–4.837)
Duan WL	2016	China	RCS	BC	NA	Transurethral bladder resection or radical cystectomy	MEG3	Down	80	NA	NA	Serum/tissue	qRT-PCR	Relative	Median (range): 57 (4–76)	RFS	Multivariate: 0.450 (0.205–0.987)
Zhao XL	2015	China	PCS	UCB	NA	Transurethral resection, partial cystectomy and radical cystectomy	SPRY4-IT1	Up	68	38	30	Tissue	qRT-PCR	Mean (3.68)	60 (Total)	OS	Multivariate: 3.716 (2.084–6.719)
Chen T	2015	China	PCS	BC	NA	Radical cystectomy	lncRNA-n336928	Up	95	44	51	Tissue	qRT-PCR	NA	60 (Total)	OS	Multivariate: 2.377 (1.007–5.610)
Fan Y	2014	China	RCS	BT	NA	NA	MALAT1	Up	95	45	50	Tissue	qRT-PCR	Median	30 (Total)	OS	Multivariate: 1.26 (0.68–2.13)
Li LJ	2014	China	PCS	BC	NA	Resection of the primary bladder cancer	GHET1	Up	80	39	41	Tissue	qRT-PCR	Median	60 (Total)	OS	Kaplan-Meier: 1.17 (0.25–5.45)
Yan TH	2014	China	PCS	BC	NA	Transurethral resection of the bladder	HOTAIR	Up	110	90	20	Tissue	qRT-PCR	Mean	60 (Total)	RFS	Multivariate: 4.712 (2.894–8.714)
He W	2013	China	RCS	BC	NA	Surgical resection	linc-UBC1	Up	102	60	42	Tissue	qRT-PCR	NA	80 (Total)	OS	Kaplan-Meier: 1.07 (0.36–3.19)

*RCS, retrospective cohort study; PCS, prospective cohort study; UBC, urinary bladder cancer; UCB, urothelial carcinoma of the bladder; MIBC, muscle-invasive bladder cancer; BTCC, bladder transitional cell carcinomas; BC, bladder cancer; NMIBC, non-muscle invasive bladder cancer; BT, bladder tumors; LncRNAs, long non-coding RNAs; Up, up-regulation; Down, down-regulation; OS, overall survival; DFS, disease free survival; CSS, cancer-specific survival; RFS, recurrence-free survival; qRT-PCR, quantitative real-time polymerase chain reaction; ISH, in situ hybridization; TCGA, The Cancer Genome Atlas; NA, not available*.

**Figure 4:**
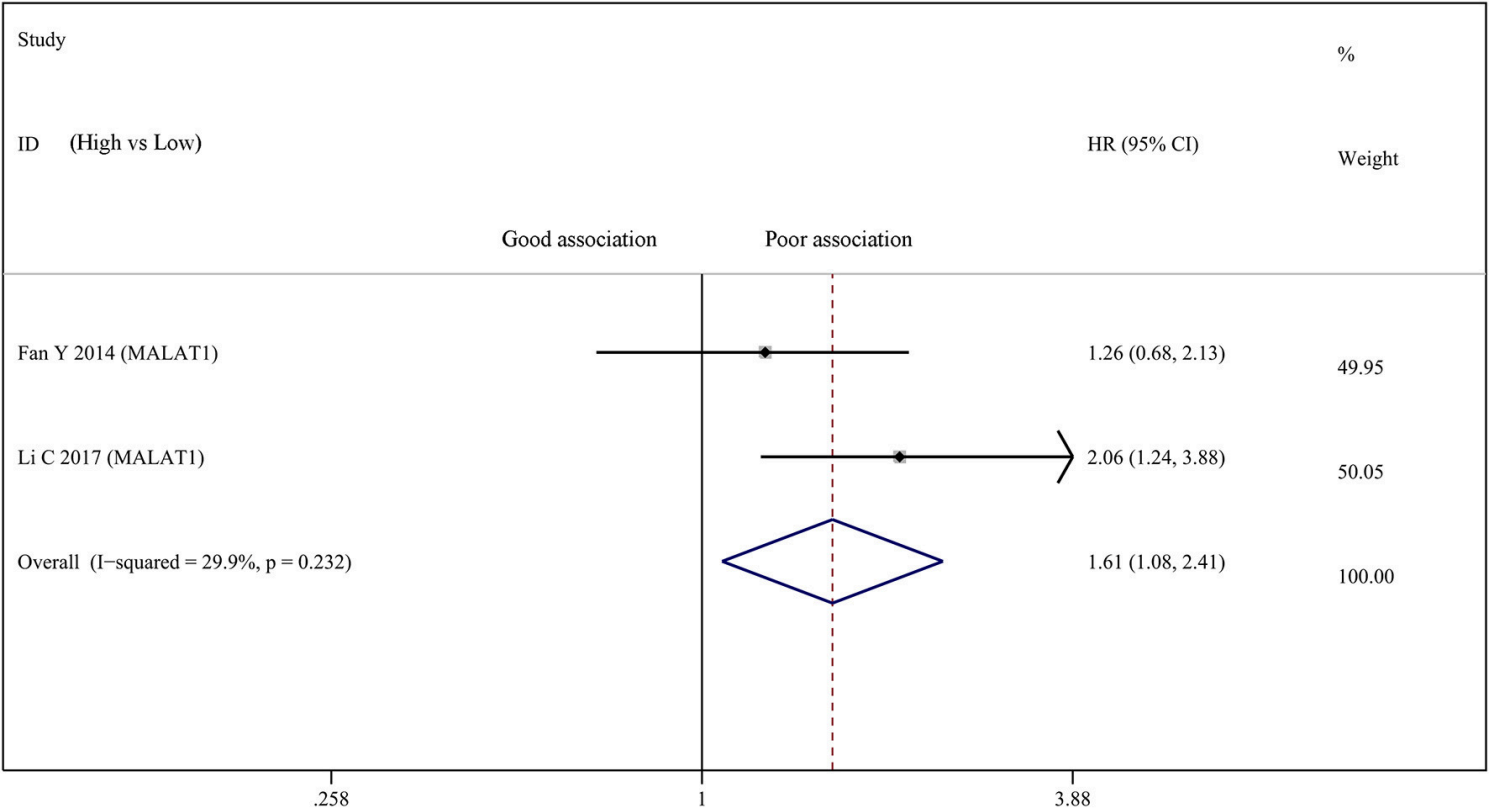
Forest plots of studies evaluating hazard ratios of up-regulated MALAT1 expression and the overall survival (OS) of bladder cancer patients.

Two studies investigated the relationship between the expression of MALAT1 and OS in a total number of 215 bladder cancer patients. Therefore, we carried out a meta-analysis on the association between abnormally expressed MALAT1 and the OS of bladder cancer patients. There was not statistically significant heterogeneity among studies (*I*_2_ = 29.9%, *p* = 0.232). After analysis using fixed effect model, our result suggested that high MALAT1 expression was significantly correlated with poor prognosis in OS (HR = 1.611; 95% CI: 1.076–2.412; *p* = 0.020) (Figure 4). Publication bias was not assessed because only two studies investigated the same lncRNA MALAT1 that were pooled into the meta-analysis.

### Diagnosis

In the diagnosis category, summary of lncRNAs used as diagnostic biomarkers of bladder cancer are presented in Table 3. Eight lncRNAs were described in the seven studies providing complete diagnostic data. The expression of SNHG16 (Duan et al., 2016), MALAT1 (Duan et al., 2016), and UCA1 (Wang et al., 2006) were up-regulated in bladder cancer tissue, while the expression of YRNA1 (Tolkach et al., 2017), YRNA3 (Tolkach et al., 2017), YRNA4 (Tolkach et al., 2017), YRNA5 (Tolkach et al., 2017), and MEG3 (Duan et al., 2016) were down-regulated. Diagnostic accuracy differed greatly between different lncRNAs tests. We found that UCA1 test had the highest sensitivity (91.5%) and specificity (96.5%) in the study conducted by Eissa et al. (Eissa et al., 2015a). Quality assessment of diagnostic studies included in the systematic review by the QUADAS tool was presented in Table S4, which showed that QUADAS scores of prognostic studies ranged from 9 to 12, indicating the high quality of included studies.

**Table 3:** Summary of lncRNAs used as diagnostic biomarkers of bladder cancer.

**Study**	**Year**	**Country**	**Study design**	**Patient population**		**TNM stage**	**LncRNAs**	**Expression**	**Sample size**		**Sensitivity (%)**	**Specificity (%)**	**AUC**	**Detected sample**	**Detection method**	**QUADAS (scores)^a^**
				**Cases**	**Controls**				**Cases**	**Controls**						
Tolkach Y	2017	Germany	Prospective	Bladder cancer	Normal urothelial tissue	NA	YRNA1 YRNA3 YRNA4 YRNA5	Down	88	30	YRNA1: 90.9 YRNA3: 88.6 YRNA4: 75.0 YRNA5: 75.0	YRNA1: 73.3 YRNA3: 80.0 YRNA4: 86.7 YRNA5: 73.3	YRNA1: 0.851 YRNA3: 0.863 YRNA4: 0.844 YRNA5: 0.715	Tissue samples	RT-qPCR	9
Duan WL	2016	China	Retrospective	Bladder cancer	Healthy and benign disease	NA	MEG3 SNHG16 MALAT1	MEG3: Down SNHG16: Up MALAT1: Up	120	120	MEG3: 70.0 SNHG16: 64.2 MALAT1: 56.7	MEG3: 75.8 SNHG16: 65.0 MALAT1: 67.5	MEG3: 0.798 SNHG16: 0.687 MALAT1: 0.640	Serum/tissue samples	RT-qPCR	10
Milowich D	2015	Belgium	Prospective	Bladder cancer	Other urological conditions	NA	UCA1	Up	70	92	70.00	70.70	NA	Urine samples	RT-qPCR	12
Eissa S	2015	Egypt	Retrospective	Bladder carcinoma	Benign urological diseases	I–III	UCA1	Up	139	45	89.2	93.3	0.966	Urine samples	RT-qPCR	11
Eissa S	2015	Egypt	Retrospective	Bladder cancer	Benign bladder lesions and age-matched normal controls	I–III	UCA1	Up	94	116	91.5	96.5	0.975	Urine samples	RT-qPCR	11
Srivastava AK	2014	India	Retrospective	Carcinoma of the urinary bladder	Healthy individuals and non-malignant disorders	NA	UCA1	Up	117	74	79.49	79.73	0.863	Urine samples	RT-qPCR	11
Wang XS	2006	China	Retrospective	Bladder cancer	Normal and other urinary tract disease	NA	UCA1	Up	94	85	80.9	91.8	0.882	Tissue samples	RT-qPCR	10

*BC, Bladder cancer; LncRNAs, long non-coding RNAs; Up, up-regulation; Down, down-regulation; AUC, area under the curve; qRT-PCR, quantitative real-time polymerase chain reaction; ^a^If a cumulative score is higher than 8, the study will be considered as low risk of bias; NA, not available*.

**Figure 5:**
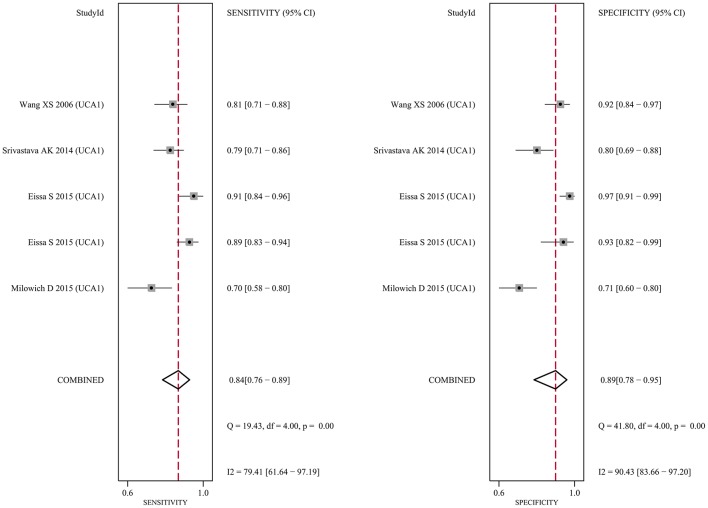
Forest plot of the sensitivity (**Left**) and specificity (**Right**) of UCA1 for the diagnosis of bladder cancer.

**Figure 6:**
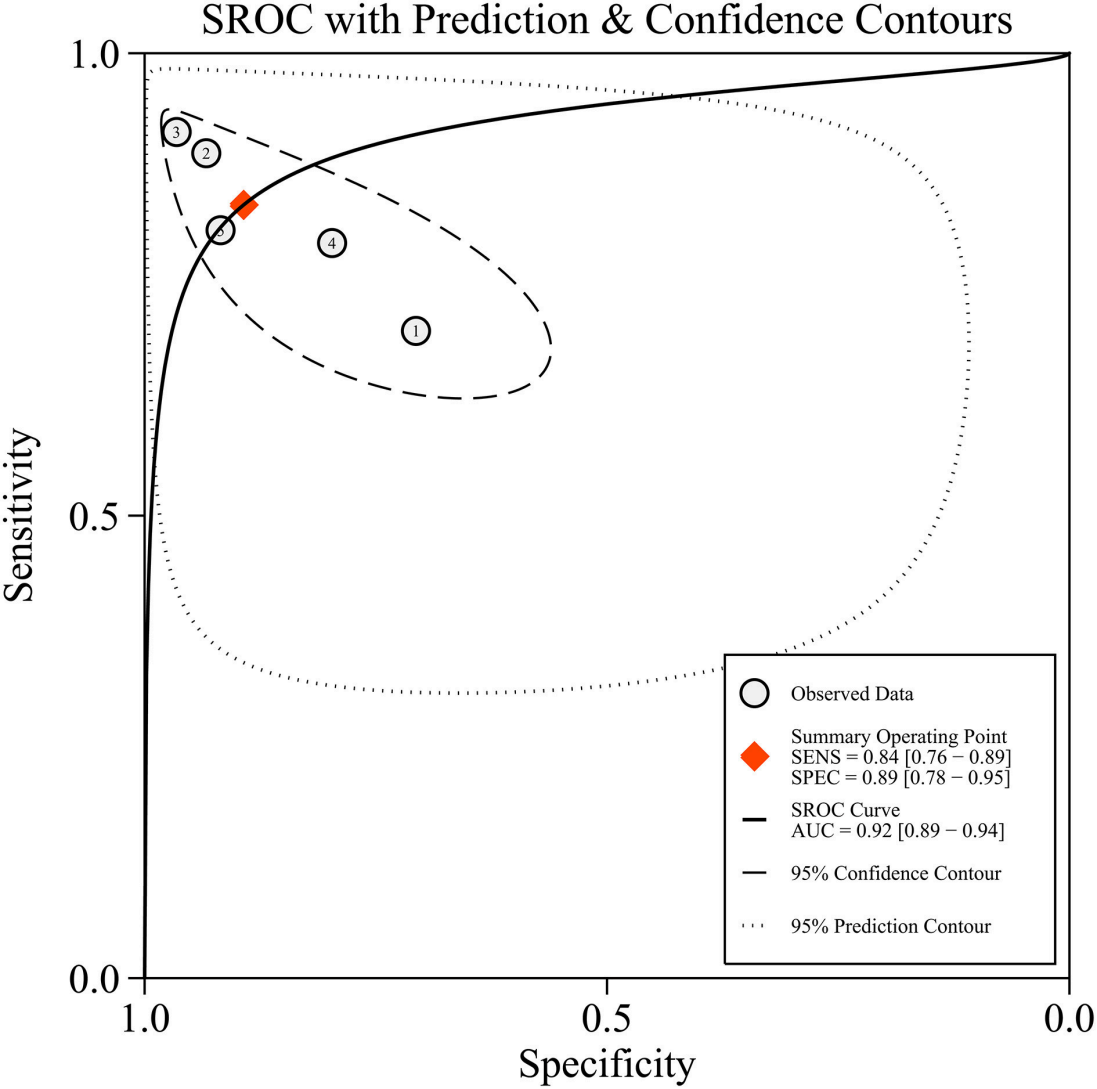
The summary receiver operator characteristic (SROC) curve based on UCA1. SECS, sensitivity; SPEC, specificity; AUC, area under the curve.

**Figure 7:**
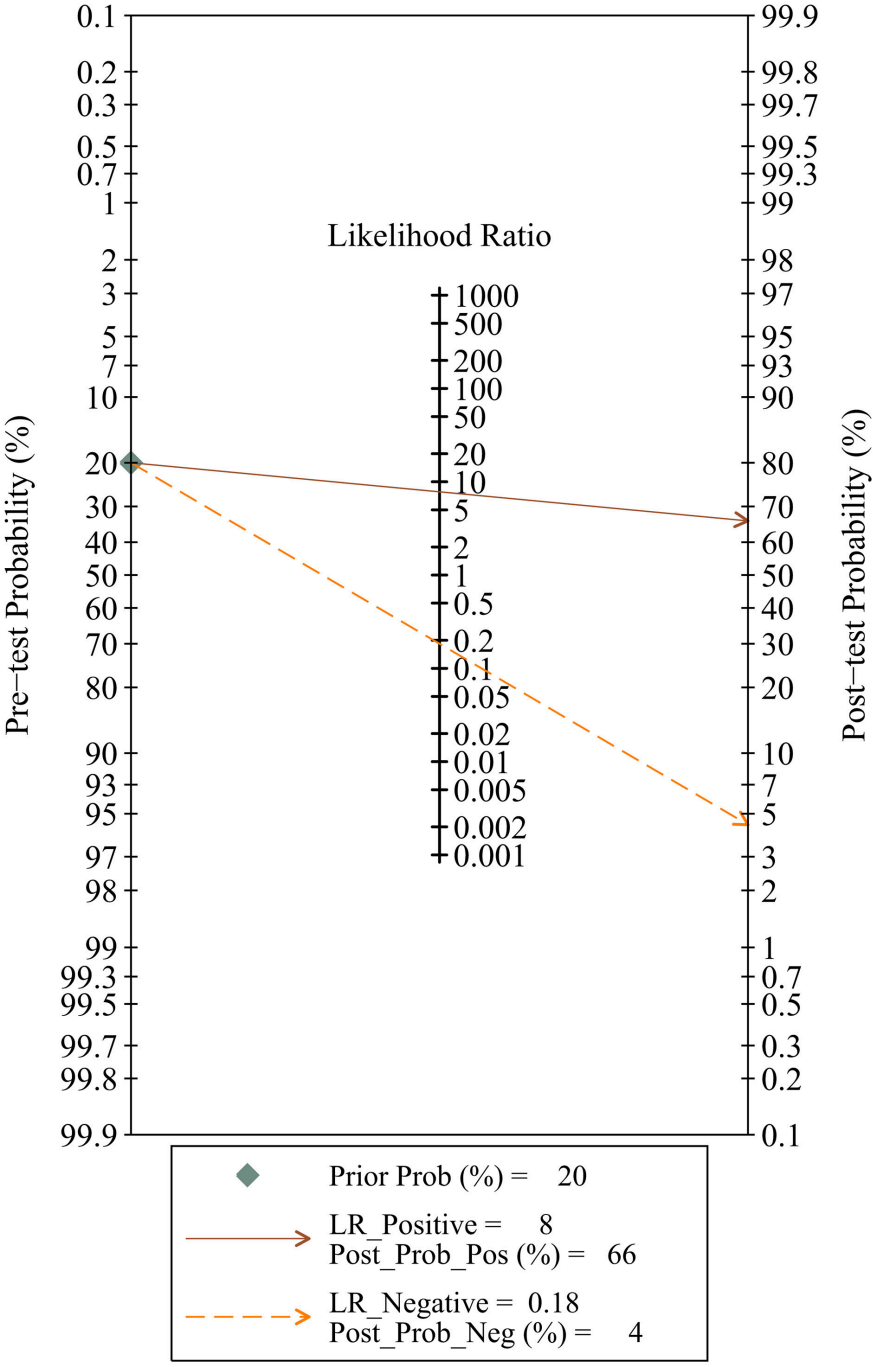
Fagan's nomogram for the calculation of post-test probability.

**Figure 8:**
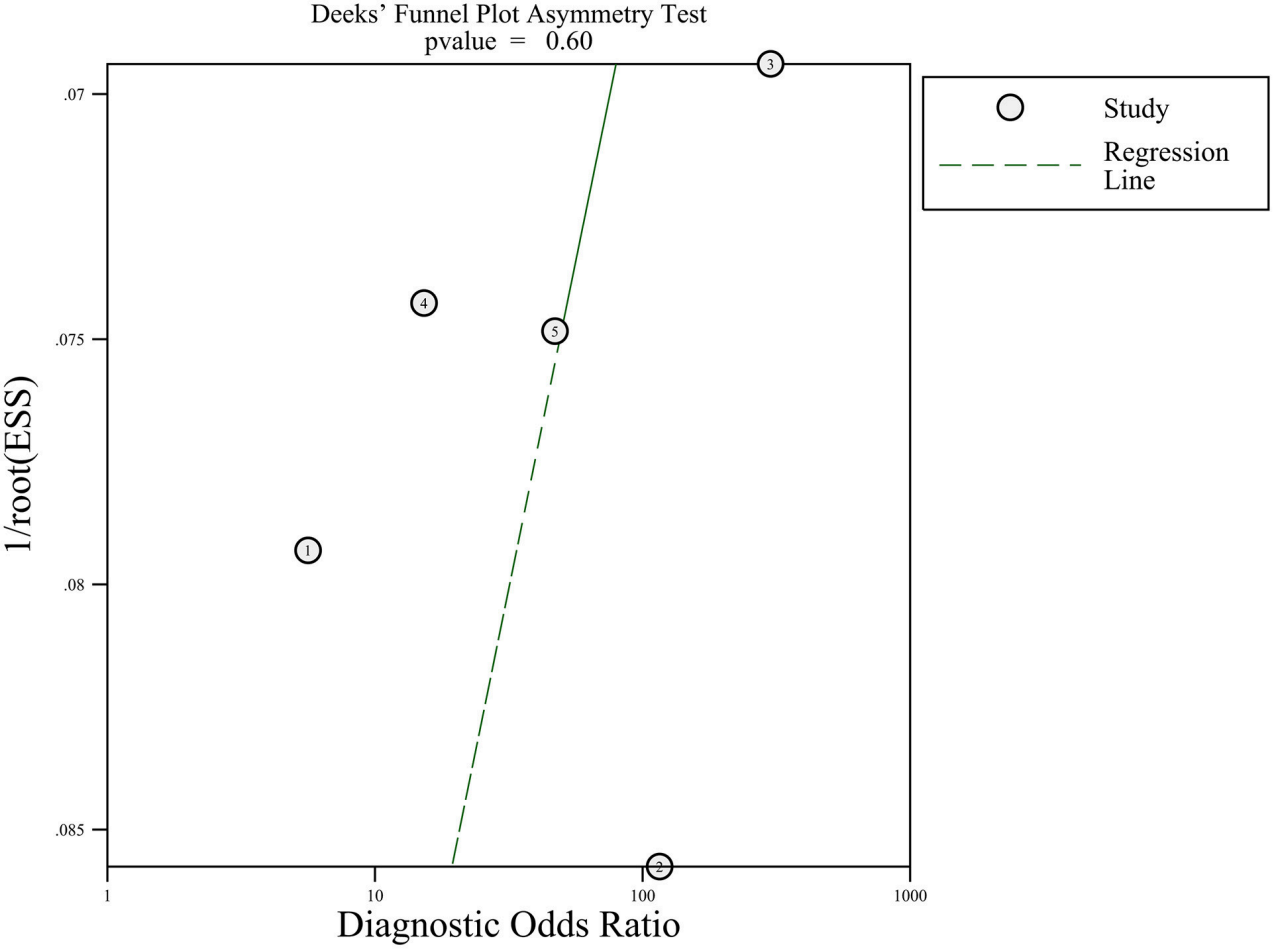
Funnel graph for the assessment of potential publication bias of the diagnostic studies.

Since five studies investigated diagnostic value of UCA1 for bladder cancer, a meta-analysis was performed based on UCA1. There was statistically significant heterogeneity in pooled sensitivity (*I*_2_ = 79.41%, *p* < 0.01) and pooled specificity (*I*_2_ = 90.43%, *p* < 0.01), then, a random-effect model was chosen for the generation of pooled indexes. The pooled sensitivity of UCA1 was 0.84 (95% CI: 0.76–0.89), specificity was 0.89 (95% CI: 0.78–0.95), PLR was 7.81 (95% CI: 3.45–17.68), NLR was 0.18 (95% CI: 0.11–0.30), and DOR was 39.65 (95% CI: 10.40–151.12). Forest plots of pooled sensitivity and specificity for UCA1 was presented in Figure 5. The AUC of SROC curve based on summary sensitivity and specificity were 0.92 (95% CI: 0.89–0.94) (Figure 6), indicating a moderate accuracy for the diagnostic test. To obtain the post-test probability, we performed a simulation of an environment that had a prevalence of 20% for bladder cancer, with base on the included studies. Incorporating this evidence in a Fagan's nomogram (Figure 7), it appeared that the positive post-test probability was 66% and the negative post-test probability 4%. Deeks' funnel plot asymmetry test was performed to check publication bias in this meta-analysis. The result indicated that no significant bias was found (*t* = −0.59; *p* = 0.598). The shape of the funnel plot was presented in Figure 8, without any evidence of obvious asymmetry. Therefore, no obvious publication bias existed in the meta-analysis of diagnostic studies.

## Discussion

Increasing evidences have indicated that abnormally expressed lncRNAs were correlated with clinical outcomes for patients with bladder cancer in recent years. Multiple lncRNAs were highlighted as potential diagnostic and prognostic biomarkers for bladder cancer and shown to be potential new targets for cancer drugs. Eissa et al. (2015b) found that there was a significant difference between bilharzial benign and malignant cases regarding urinary lncRNA-UCA1 expression, and suggested that UCA1-nanoassay was a valid test for direct detection of urine UCA1 for bladder cancer detection. Fan et al. (2014) revealed that MALAT1 level was higher in primary tumors that subsequently metastasized than those in non-metastatic bladder cancer, and suggested that MALAT1 inhibition may represent a promising therapeutic option for suppressing bladder cancer progression. While Li et al. (2017) demonstrated that high tumor stage, positive lymph nodes, and high MALAT1 expression were independent prognostic indicators for OS of bladder cancer patients, and suggested that high MALAT1 expression could be considered as a potential therapeutic target of bladder cancer. However, most studies examining the clinical values of aberrantly expressed lncRNAs was limited by relatively small sample size or single lncRNA, which may result in inconsistent biological conclusions. Therefore, we performed a comprehensive systematic review and meta-analysis to systematically evaluate the clinical values of various lncRNAs in bladder cancer.

In the present systematic review, we investigated the relationship between multiple lncRNAs and clinicopathological parameters of bladder cancer. Most of the included studies suggested that multiple lncRNAs might be used as potential biomarkers of histological grade, TNM stage, tumor stage T, and lymph node metastasis. Many lncRNAs were identified in multiple different studies but only 2 (MALAT1 and XIST) were found to be studied in more than one study. For the MALAT1, Li et al. (2017) reported that high expression of MALAT1 was closely associated with higher probability of lymph node metastasis; but Fan et al. (2014) did not find statistically significant association between increased MALAT1 expression and lymph node metastasis. Pooled result showed that high expression of MALAT1 was significantly associated with lymph node metastasis, suggesting that MALAT1 can serve as a valuable biomarker for predicting lymph node metastasis status. For the XIST, high expression of XIST was related to larger tumor size and higher TNM stage after we pooled OR, suggesting that XIST can serve as a valuable biomarker for predicting tumor size and TNM stage in patients with bladder cancer. However, we did not provide enough information about bladder cancer patients. Some studies reported that patients were treated by surgery only and did not receive radiotherapy, chemotherapy, or other therapy before surgery; others did not mention treatment. Clinicopathological data such as gender, age, histological grade, TNM stage, or lymph nodal status were obtained at the same time. TNM stage was reported only in some studies and ranged from 0 to IV. LncRNAs expression was detected using by quantitative real-time PCR in tissue sample which were obtained and immediately frozen at the same time. Then study evaluated the association between lncRNAs and clinicopathological parameters. So whether the patients can be cured was not reported in the most studies included.

We explored the prognostic role of multiple lncRNAs in bladder cancer. For OS, we found that the increased expressions of 11 lncRNAs were related to poor prognosis in bladder cancer, while the decreased expressions of 6 lncRNAs were related to poor prognosis. Among them, SPRY4-IT1 (Zhao X. L. et al., 2015) exhibited the highest HR of 3.72, while NBAT1 (Du et al., 2017) exhibited the lowest HR of 0.41. Meta-analysis of different lcRNAs with prognosis was not performed, because we thought that meta-analysis of random lcRNAs doesn't make sense on a scientific level. However, MALAT1 was investigated in two studies (Fan et al., 2014; Li et al., 2017), so we carried out a meta-analysis on the association between abnormally expressed MALAT1 and the OS of bladder cancer patients. Our result suggested that high expression of MALAT1 was significantly correlated with poor prognosis in OS among patients with bladder cancer. A previous meta-analysis conducted by Tian and Xu (2015) reported that MALAT1 expression was an independent prognostic marker for OS in patients with cancer using univariate and multivariate analyses, those findings in consist with our results. Therefore, high MALAT1 expression can serve as an independent prognostic factor for OS of bladder cancer patients and can be considered as a potential therapeutic target of bladder cancer.

Diagnostic accuracy of multiple lncRNAs tests was explored in the present systematic review. Different lncRNAs tests differed in their sensitivity and specificity. Five studies investigated diagnostic value of lncRNA UCA1 for bladder cancer, but diagnostic accuracy differed greatly among these studies. Meta-analysis is a method of summarizing discrepant data on the accuracy of diagnostic tests. So a meta-analysis was performed based on UCA1. As a result, the overall pooled sensitivity and specificity of UCA1 for bladder cancer were 0.84 and 0.89, respectively, along with an AUC value of 0.92, suggesting that the diagnostic accuracy of UCA1 was moderate. However, there was statistically significant heterogeneity in pooled sensitivity and pooled specificity. We supposed that the patient populations, sample size, and the different cut-off value might be the potential source. But data were not enough to illustrate main source of heterogeneity. Thus, a meta-regression analysis is urgently needed to assess how study-specific attributes caused heterogeneity. The DOR shows the correlation between diagnostic efficiency and the disease, which has better discriminatory test performance with an extremely higher value (Glas et al., 2003). In current study, the DOR value was calculated to be 39.65, suggesting a moderate diagnostic accuracy of UCA1 for bladder cancer diagnosis. In addition, a lower NLR and a higher PLR values show a better diagnostic performance. In this study, the pooled PLR and NLR values were calculated to be 7.81 and 0.18 for UCA1, respectively, also suggesting a moderate diagnostic accuracy. These data suggested that UCA1 expression test showed a moderate diagnostic accuracy for bladder cancer. Therefore, UCA1 can be considered as a potential biomarker to assist in the diagnosis of bladder cancer. The diagnostic significance of UCA1 expression in bladder cancer has been investigated by meta-analysis in recent studies (Wang Z. et al., 2017; Zhen et al., 2017). Compared with a previous meta-analysis (Wang Z. et al., 2017), we found that there was similar sensitivity, but there was slightly higher specificity, DOR, and AUC in our results. The different in results may be due to the fact that non-English papers were excluded from our meta-analysis.

There are several limitations in our systematic review. Firstly, most of the included studies were conducted in Chinese populations, so the conclusion of this study might not be extended to all populations. Secondly, mutiple lncRNAs were used to evaluate the clinicopathological parameters, prognosis, and diagnosis of bladder cancer, so we may overestimate the clinical values of single lncRNA. Thirdly, HR and its 95% CI were extracted from Kaplan–Meier survival curves in seven studies, which may be less reliable than those directly acquired from survival data. Finally, although our searches were extensive and were not limited by language, language bias should not be completely avoided because of all included studies written in English.

In conclusion, systematic review elaborated that abnormal lncRNAs expression can serve as potential markers for prognostic evaluation in bladder cancer patients. More importantly, the diagnostic meta-analysis concluded that abnormally expressed UCA1 can function as potential diagnostic markers for bladder cancer. However, most lncRNAs were investigated in a single study, whose results were not enough to illustrate the clinical value of lncRNAs completely. So larger-size and higher-quality studies need to be conducted to validate the clinical value of single lncRNA in patients with bladder cancer.

## Author contributions

GS and QH designed this study. GS, QH, and JW participated in study selection and data extraction. GS, QH, and JW performed statistical analysis. GS and QH wrote and reviewed the manuscript.

### Conflict of interest statement

The authors declare that the research was conducted in the absence of any commercial or financial relationships that could be construed as a potential conflict of interest. The reviewer VC and handling Editor declared their shared affiliation.
