# Supplementary material for: Clinical Values of Long Non-coding RNAs in Bladder Cancer: A Systematic Review
Source: Front Physiol. 2018 May 30;9:652. doi: 10.3389/fphys.2018.00652 (PMC5988895; doi:10.3389/fphys.2018.00652)
Supplement: Supplementary file 2 [file Table_2.DOC]

| **TABLE S2｜**The detailed search strategies and results. | | | |
| --- | --- | --- | --- |
| **Database** | **Last Update** | **Search Strategy** | **results** |
| PubMed | December 21, 2017 | #1 Search ((((long non-coding RNA) OR lncRNA) OR lincRNA) OR long ncRNA) OR long intergenic non-coding RNA Sort by: Best Match  #2 Search bladder Sort by: Best Match  #3 Search ((((((((cancer) OR carcinoma) OR neoplasm) OR tumor) OR tumors) OR tumour) OR tumours) OR malignancy) OR metastasis Sort by: Best Match  #4 #1 AND #2 AND #3 | 185 |
| Embase | December 21, 2017 | #1 ‘long non-coding RNA’ OR ‘lncRNA’ OR ‘lincRNA’ OR ‘long ncRNA’ OR ‘long intergenic non-coding RNA’  #2 ‘bladder’  #3 ‘cancer’ OR ‘carcinoma’ OR ‘neoplasm’ OR ‘tumor’ OR ‘tumors’ OR ‘tumour’ OR ‘tumours’ OR ‘malignancy’ OR ‘metastasis’  #4 #1 AND #2 AND #3 | 215 |
| Web of Science | December 21, 2017 | TOPIC: (‘long non-coding RNA’ OR ‘lncRNA’ OR ‘lincRNA’ OR ‘long ncRNA’ OR ‘long intergenic non-coding RNA’) AND TOPIC: (‘bladder’) AND TOPIC: (‘cancer’ OR ‘carcinoma’ OR ‘neoplasm’ OR ‘tumor’ OR ‘tumors’ OR ‘tumour’ OR ‘tumours’ OR ‘malignancy’ OR ‘metastasis’) | 337 |
